# Revisiting the Spectroscopy of Water Dimer in Jets

**DOI:** 10.1021/acs.jpclett.0c03001

**Published:** 2021-02-04

**Authors:** Iker León, Raúl Montero, Asier Longarte, José A. Fernández

**Affiliations:** † Grupo de Espectroscopía Molecular (GEM), Edificio Quifima, Unidad Asociada CSIC, Universidad de Valladolid, 47005 Valladolid, Spain; ‡ SGIKER Laser Facility, University of the Basque Country (UPV/EHU), Barrio Sarriena s/n, Leioa 48940, Spain; § Department of Physical Chemistry, Faculty of Science and Technology, University of the Basque Country (UPV/EHU), Barrio Sarriena s/n, Leioa 48940, Spain

## Abstract

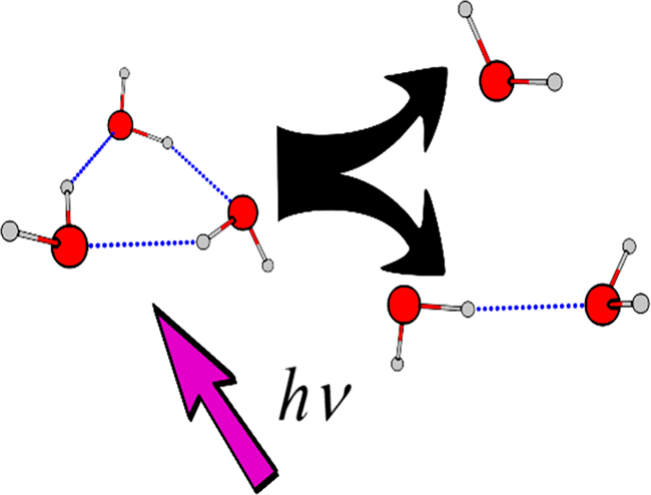

Laser spectroscopy in jets is one of the main sources of structural data from molecular aggregates. Consequently, numerous and sophisticated experimental systems have been developed to extract precise information, which is usually interpreted in the light of quantum mechanical calculations. However, even with the most sophisticated experiments, it is sometimes difficult to interpret the experimental results. We present here the example of water dimer and how after almost 70 years, the assignment of its mass-resolved IR spectrum still generates controversy that extends toward the mechanism of ionization of water aggregates.

The combination of laser spectroscopy with supersonic expansions has proven to be a powerful method to obtain information on the structure of molecules and aggregates formed by noncovalent interactions.^1^ The cooling conditions of the expansion provide a suitable environment for the efficient formation of aggregates, while the high photon flux of the laser boosts the detection of the species formed. Introduction of the REMPI (resonance-enhanced multiphoton ionization) technique^2^ in tandem with mass spectrometers brought in two key features: the sensitivity of ion detection and mass resolution. Since then, REMPI methods have been applied to a myriad of systems, starting from the simplest aromatic rings to large aggregates and biological molecules.^3,4^ When REMPI is used in combination with IR spectroscopy, in what has been termed double-resonance techniques, it enables the extraction of relevant structural information.^5^ Certainly, IR-REMPI adds mass resolution to the traditional IR spectroscopy, enabling the disentanglement of the IR spectra of each isomer of a given stoichiometry from the complex mixture of species formed in the supersonic expansions. The technique has evolved into sophisticated setups that use up to four lasers to tackle the spectroscopy of even the most complicated system.^6−8^ The only condition that the system must fulfill to be studied by this method is to have a chromophore with an optically accessible electronic state with a reasonably long lifetime (longer than a few picoseconds). This condition is necessary for the multiphoton absorption to take place in a resonant manner, substantially increasing the ion yield and the final signal.

Despite the many molecules that fulfill such conditions, there are paradigmatic systems that are forbidden to the IR-REMPI combination. Therefore, there have been many techniques developed to take the mass-resolved IR spectroscopy to the realm of the systems without a chromophore, although the main principle behind them is to find a way to circumvent the stepping stone of the intermediate electronic state.^9−11^ An additional drawback that limits the extraction of mass-selective IR spectra is the appearance, upon ionization, of cations that result from the fragmentation of the initially formed cluster ions. The coexistence in the beam of species of different size and the typical reduction in the ionization energy threshold that accompanies cluster growth make avoiding this fragmentation difficult. Thus, the IR spectra of the targeted species may appear in different mass-channels, strongly complicating the assignment of the spectra. In this context, the search for ionization sources^12−18^ and ionization schemes^6−8^ able to minimize and/or identify fragmentation patterns has been another key element in the evolution of the instrumentation in the field.

One of the subjects that has guided such developments has been the study of water aggregates and in particular the simplest one: the water dimer. It represents the paradigm of a system attached by a pure O–H···O hydrogen bond, and it is a key step in the understanding of the behavior of water.^19^ Characterization of its structure goes back to the studies by Pimentel’s group in the 1950s in N_2_ matrices^20^ and has motivated numerous studies using a collection of spectroscopic techniques, although IR optical spectroscopy has been the main tool applied to probe the structure of the neutral cluster. For an excellent review on the matter, see ref (19). Despite its small size, the dimer has a complex spectroscopy due to the presence of three tunneling pathways that lead to several splittings of the energy levels. Furthermore, the sticky nature of water makes it difficult to control the formation of the aggregate of choice, and therefore, dimer formation is usually accompanied by the presence of trimers, tetramers, or even larger aggregates. Ionization of water clusters avoiding fragmentation or secondary reactions in the ion is far from being a simple matter. Upon ionization, the (H_2_O)_*n*_^+^ ions undergo very fast intracluster reactions. The most favored process involves the formation of protonated species accompanied by the loss of ^•^OH radical and neutral water molecules:^21−25^
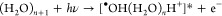
1
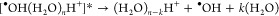
2In fact, it has been postulated that poor Franck–Condon factors between the neutral and ionic states preclude the observation of cluster ions larger than the dimer, with the protonated species being the only ions observed.^21,22^ All these facts have resulted in a history of assignments and reassignments of the IR spectrum of water dimer.

Page *et al.*(26) and Coker *et al.*(27) published the first studies on the IR spectroscopy of water clusters in supersonic expansions. Using electron bombardment as ionizing source in combination with a quadrupole mass detector, the former authors obtained what was considered the first gas-phase IR spectrum of water dimer. The spectrum, recorded in the H_3_O^+^ channel, exhibited, among other relevant features, a band at 3545 cm^–1^ that was assigned to the stretch of the donor OH. As we will describe below, the assignment of this peak to the dimer, which was reassigned to the trimer band by subsequent works,^13,28−30^ is essential in the development of the actual knowledge about the structure of the dimer and larger clusters.

Coker *et al.*(27) used a color center laser and a cryogenic bolometer as a detector to record the IR spectrum of the dimer. Despite the careful design of the experiment, the authors were not able to avoid interference from larger clusters in the spectrum of what they took as the water dimer. On the basis of previous reports^26,31^ and in their theoretical analysis, they also assigned the 3532 cm^–1^ band to the stretch of the donor OH. Several years later, the same group published a second paper on the spectroscopy of water dimer, using higher resolution.^32^ Still, they maintained the assignment of the band at 3532 cm^–1^ to the water dimer, despite it being less resolved than the other bands in the spectrum. A substantially more sophisticated experimental setup allowed Huisken *et al.* to correct the established assignment of the dimer’s IR spectrum.^28^ They used a crossed molecular beam apparatus, in which a secondary rare gas beam was employed to disperse the cluster beam. Detecting the scattered species with different angles, a sort of mass selectivity could be achieved. Although they were not able to avoid interference from the trimer and tetramer, this technique allowed the authors to identify a relatively weak band in the spectrum at 3601 cm^–1^ as the stretch of the donor OH and the band at 3532 cm^–1^ as being due to the trimer.

A different approach was used by Zwier’s group, which used benzene to nucleate water clusters.^34^ In theory, the aromatic ring should introduce only subtle perturbations in the structure of the aggregates. Furthermore, it acts as a chromophore, enabling the use of IR-REMPI spectroscopy. Despite this latter advantage, the authors reported extensive cascade fragmentation, precluding a clear assignment of the spectral features. Once more, the experimental observations favored the assignment of a band at 3550 cm^–1^ as the stretch of the hydrogen-bonded OH, although the authors warned that such assignment should be taken with caution because of the existence of fragmentation. The same group recently revisited the spectroscopy of benzene–water aggregates, conducting new experiments with an improving *s*/*n* ratio and using sophisticated quantum mechanical computations to rationalize the results.^35^ Unfortunately, the authors did not offer explicit values for the vibrations, but it is clear from the experimental spectrum that the aromatic ring induces non-negligible perturbations in the IR spectrum of water dimer.

The first work to fully establish the frequencies of the four OH stretching bands of the dimer was published by Vilesov’s group.^29^ The water clusters were formed in He droplets, and their spectra were registered in the 3580–3820 cm^–1^ region. Although the authors did not extend their measurements to the region of the controversial band at ∼3540 cm^–1^, they were able to detect and assign, with the help of calculations, all the OH stretches of the dimer. Remarkably, the excellent *s*/*n* ratio achieved in the experiment allowed them to report for the first time the position of the weak symmetric stretch of the acceptor water molecule (see Table 1). The splittings due to the several tunneling effects in water dimer cause the antisymmetric acceptor stretch to appear as several well-resolved lines at the blue-end of the spectrum. From those lines, the authors determined the position of the band origin to be at 3748.6 cm^–1^. The spread of the intensity of this vibration among several lines may have induced an incorrect assignment of that portion of the spectrum of water dimer.

**Table 1:** Values (in cm^–1^) Reported by Several Authors for the Vibrations of Water Dimer and Trimer

	proposed assignments							
mode	Coker *et al.*^a^	Page *et al.*^b^	Huisken *et al.*^c^	Zhang *et al.*^d^	Leon *et al.*^e^	this work	Kuyanov-Prozument *et al.*^f^	Pribble *et al.*^g^
dimer								
donor stretch (free OH)	3730 ± 3	3730		3732			3730.1	
acceptor asymmetric stretch	3722 ± 3	3714	3735	3764, 3780	3730, 3732		3748.6	3722, 3708
acceptor symmetric stretch	3600 ± 3	3600		3603	3670^h^		3654.3	3608^i^
donor stretch (bonded OH)	3532 ± 3	3545	3601	3549, 3537	3601		3597.4	3550^j^
trimer	3400 ± 5		3533			3543		3550, 3508, 3423^i^
trimer	3357 ± 3							
tetramer			3416			3400		3461, 3427, 3367^j^
donor bend overtone	3215 ± 5							
acceptor bend overtone	3170 ± 5	3186						
technique	IR with a cryogenic bolometer	IR + electron bombardment	crossed molecular beam	IR-tunable VUV	IR^ns^–IR^fs^	IR^ns^–IR^fs^	He droplets	IDIRS in benzene–water aggregates

a Ref (27).

b Ref (26).

c Ref (28).

d Ref (33).

e Ref (13).

f Ref (29).

g Ref (34).

h Computed value.

i π-bonded OH stretch.

j According to the authors, these values may be affected by fragmentation.

The next band to the red at 3730.1 cm^–1^ was assigned as the free OH stretch of the donor water molecule. Interestingly, the authors also reported a band due to larger water clusters at ∼3718 cm^–1^ (value estimated from the figure in their paper). Following to the red, a very weak feature appeared at 3654 cm^–1^, corresponding to the symmetric stretch of the acceptor water molecule. Next to the red, the donor OH stretch was assigned to a prominent band at 3597 cm^–1^, in full agreement with Huisken *et al.*(28) Comparison of these values with those from other techniques highlights that the He atoms introduce a small shift in the position of the bands, certainly smaller than the perturbation introduced by the benzene ring in the work from Zwier’s group.

We added to the field of the spectroscopy of water dimer by introducing several years ago a new technique consisting of the combination of femtosecond and nanosecond lasers to obtain the mass-resolved IR spectrum of molecules without a chromophore.^13−16^ The fundamentals behind this technique were that using 800 nm fs pulses with intensities on the edge of the barrier-suppression regime, it was possible to ionize the molecules while minimizing the fragmentation by fine-tuning the intensity of the probe laser and the conditions of the expansion. In those cases in which some fragmentation persisted, it was possible to introduce a second nanosecond IR laser to record isomer-specific mass-selected IR spectra. Thanks to this new experimental setup, we recorded the spectrum in Figure 1a, which was collected directly in the (H_2_O)_2_^+^ channel, avoiding interference from larger species. Therefore, it may be taken as the first report on the mass-resolved IR spectrum of the water dimer without contribution from larger aggregates.^13^ The spectrum contains only two clear bands and some additional features to the blue, instead of the four bands one would expect for water dimer, namely: H-bonded and free OH stretches of the donor molecule and symmetric and antisymmetric stretches of the acceptor molecule. The assignment adopted was in perfect agreement with that from Kuyanov-Prozument *et al.*(29) and was based on the absence of the band corresponding to the symmetric stretch of the acceptor molecule, for which the calculations conducted at the M06-2X/6-311++G(d,p) level predicted a very low intensity. Such an absence may be one of the sources of confusion that led several authors to incorrectly assign the band at 3601 cm^–1^ as the symmetric stretch of the acceptor molecule. It is important to note that the lower resolution of the spectrum in Figure 1 and the reduced *s*/*n* ratio compared with that of Vilesov’s group,^29^ precluded a more accurate assignment of the antisymmetric stretch of the acceptor water molecule.

**Figure 1:**
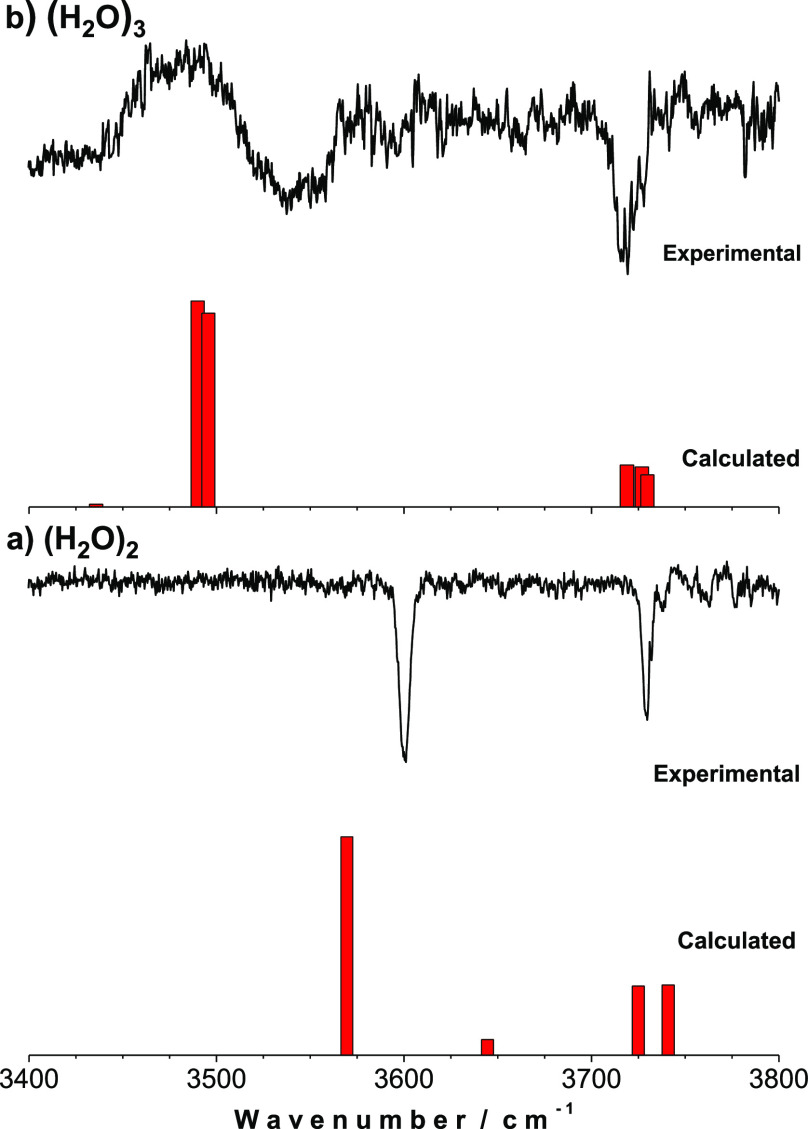
Comparison between the experimental spectra of water dimer (a) and trimer (b) and the frequencies predicted at the M06-2X/6-311++G(d,p). The spectra were recorded in their corresponding (H_2_O)_2_^+^ and (H_2_O)_3_^+^ mass-channels, using an 800 nm fs probe laser and an IR ns depopulation laser. Scaling factor 0.942. The spectrum of water dimer was adapted from ref (13).

As mentioned above, probably the main controversial aspect in the interpretation of the IR spectrum of water dimer is the identity of the band’s system located by the different studies in the 3532–3550 cm^–1^ interval, which has been attributed alternatively to the donor OH stretch of the dimer^26,27,34^ or to fragmentation from larger clusters. The attribution of a peak in the 3529–3532 cm^–1^ interval to the trimer has been proposed by a number of authors employing different techniques: gas-phase IR spectroscopy,^13,19,28,30,36,37^ cold matrices,^2,38,39^ or He droplets.^29,40^ There is a compilation of the assignments proposed for that band in ref (37). This feature is often accompanied by additional peaks in the region that have been associated to the trimer or larger clusters. Our own data also show that the double band located at ∼3543 cm^–1^ was present in the spectrum recorded in the mass channel of the trimer (Figure 1b). Summarizing, the accepted view in the field is that the IR spectrum of the dimer OH stretches extends from the bonded donor stretch at ∼3600 cm^–1^ to the antisymmetric acceptor stretch features at ∼3750 cm^–1^, while the bands observed in the 3529–3550 cm^–1^ are associated to the trimer or larger clusters.

In a recent paper, Zhang *et al.*(33) revisited the IR spectroscopy of the water dimer formed in a He supersonic expansion, by means of a double-resonance technique that employed tunable VUV radiation from a free-electron laser (FEL) as ionization source. The use of one VUV photon ionization has already been explored by several authors,^12,17,21,22,24,25,41−43^ as the first ionization potential of molecules and their clusters generally lie in this energy region. Compared to traditional REMPI, this method does not require the presence of intermediate states, making it suitable for virtually any molecular system. As an additional advantage, the use of tunable VUV radiation permits, in principle, to precisely reach the ionization threshold, minimizing the appearance of dissociation processes in the formed ions.

On the basis of these ideas, by carefully adjusting the conditions of the expansion and the VUV source (wavelength and pulse energy), the authors were able to record the IR spectrum of the dimer directly in the (H_2_O)_2_^+^ channel. The assignment of the dimer’s OH stretches by Zhang *et al.*(33) assuming the absence of fragmentation followed that proposed by Coker *et al.*,^27^ Page *et al.*,^26^ and Pribble *et al.*,^34^ attributing the doublet at ∼3543 cm^–1^ to the donor H-bonded OH, and consequently, the band at the ∼3600 cm^–1^ was associated with the symmetric stretch of the acceptor molecule, as in the works by the above-mentioned authors. This interpretation is in clear contradiction with the most recent studies reported on the dimer and trimer that have been described above (Table 1). However, in addition, the result itself challenges some well-established ideas in the field.

First, the authors were able to record the IR spectrum of the dimer directly in the (H_2_O)_2_^+^ mass channel, and their mass spectra also show the unprotonated trimer ion (H_2_O)_3_^+^. Furthermore, in a recent work employing the same method, they reported the IR spectra of (H_2_O)_*n*_^+^ (*n* = 3–6), recorded in their own mass channels.^44^ If this result is confirmed, it will be the first time that water clusters larger than the dimer are detected in their own mass channel, using a VUV ionization source. The general consensus in the field^17,21,22,37,45^ dictates that the vertical ionization brings the cluster to a region of the potential energy surface of the ion above the barrier for reaction 1, dissociating a water molecule into a proton and an ^•^OH radical. The calculations demonstrate that the ^•^OH radical forms weaker hydrogen bonds than water, and therefore, it is the most weakly attached moiety and the first one to be evaporated by the extra energy supplied by the ionization process, leaving behind a protonated cluster.^45,46^

Such ideas are further supported by the experiments on ionization of water aggregates embedded in Ar clusters by Washida and co-workers.^21,22^ They demonstrated that under such conditions it was possible to detect the parent H_2_O^+^ ion. The authors argued that the extra energy delivered during ionization is released by evaporation of Ar atoms, cooling the water aggregate and enabling the detection of the parent ions.^47^ Thus, except for the dimer, experiments similar to those reported by Zhang *et al.* had inexorably failed to detect unprotonated water clusters.^25,48^ The results presented by Zhang *et al.* challenge those ideas and ask for a reinvestigation of the subject.

Second, the presence in the dimer’s IR spectrum recorded by Zhang *et al.* of features (the doublet at ∼3549 cm^–1^) that are associated with larger clusters imply the formation of unprotonated (H_2_O)_2_^+^ fragments from (H_2_O)_*n*>2_^+^ ions. Although this channel should be accessible for the 98.1 nm radiation employed (12.64 eV),^25^ which is 0.9 eV above the adiabatic ionization threshold estimated for the trimer, it should be suppressed by the thermodynamically favored formation of protonated dimers or monomers according to reaction 2. Once more, the evidence presented in ref (33) demands a reinvestigation of the reaction mechanisms proposed for the ionization of water aggregates.

In summary, the data presented in this Viewpoint demonstrate that even small and apparently simple systems such as water dimer and trimer still hold relevant and deep questions. Perhaps one of the problems associated with revealing the true mechanism underlying these fundamental processes is that there is no single technique that can give the answer, and therefore, it is necessary to accumulate evidence from different experimental techniques, each of them adding a small piece to the puzzle. Still, it is very often difficult to fit those pieces in place. Understanding the whole picture can be accomplished only through the work of multiple research groups.

## References

[ref1] Hobza P.; Muller-Dethlefs K. In Non-Covalent Interactions; RCS Publishing: Cambride, 2010.

[ref2] Bouteiller Y.; Perchard J. P. The vibrational spectrum of (H_2_O)_2_: comparison between anharmonic ab initio calculations and neon matrix infrared data between 9000 and 90 cm^–1^. Chem. Phys. 2004, 305, 1–12. 10.1016/j.chemphys.2004.06.028.

[ref3] Lubman D. M.; Kronick M. N. Mass spectrometry of aromatic molecules with resonance-enhanced multiphoton ionization. Anal. Chem. 1982, 54, 660–665. 10.1021/ac00241a014.

[ref4] Schermann J. P. In Spectrocopy and modelling of biomolecular building blocks, 1st ed.; Elsevier: Amsterdam, 2008.

[ref5] Zwier T. S. The Spectroscopy of Solvation in Hydrogen-Bonded Aromatic Clusters. Annu. Rev. Phys. Chem. 1996, 47, 205–241. 10.1146/annurev.physchem.47.1.205.

[ref6] Weiler M.; Bartl K.; Gerhards M. Infrared/ultraviolet quadruple resonance spectroscopy to investigate structures of electronically excited states. J. Chem. Phys. 2012, 136, 114202–114206. 10.1063/1.3693508. 22443757

[ref7] León I.; Usabiaga I.; Millán J.; Cocinero E. J.; Lesarri A.; Fernández J. A. Mimicking anesthetic-receptor interactions in jets: the propofol-isopropanol cluster. Phys. Chem. Chem. Phys. 2014, 16, 16968–16975. 10.1039/C4CP01702A. 25005780

[ref8] León I.; Cocinero E. J.; Millán J.; Rijs A. M.; Usabiaga I.; Lesarri A.; Castaño F.; Fernández J. A. A Combined Spectroscopic and Theoretical Study of Propofol·(H_2_O)_3_. J. Chem. Phys. 2012, 137, 074303 10.1063/1.4743960. 22920116

[ref9] Hu Y. J.; Fu H. B.; Bernstein E. R. Infrared plus vacuum ultraviolet spectroscopy of neutral and ionic methanol monomers and clusters: New experimental results. J. Chem. Phys. 2006, 125, 154306 10.1063/1.2357953. 17059254

[ref10] Yatsyna V.; Mallat R.; Gorn T.; Schmitt M.; Feifel R.; Rijs A. M.; Zhaunerchyk V. Competition between folded and extended structures of alanylalanine (Ala-Ala) in a molecular beam. Phys. Chem. Chem. Phys. 2019, 21, 14126–14132. 10.1039/C9CP00140A. 30869702

[ref11] Yatsyna V.; Mallat R.; Gorn T.; Schmitt M.; Feifel R.; Rijs A. M.; Zhaunerchyk V. Conformational Preferences of Isolated Glycylglycine (Gly-Gly) Investigated with IRMPD-VUV Action Spectroscopy and Advanced Computational Approaches. J. Phys. Chem. A 2019, 123, 862–872. 10.1021/acs.jpca.8b10881. 30608157

[ref12] Fu H. B.; Hu Y. J.; Bernstein E. R. IR+vacuum ultraviolet (118 nm) nonresonant ionization spectroscopy of methanol monomers and clusters: Neutral cluster distribution and size-specific detection of the OH stretch vibrations. J. Chem. Phys. 2006, 124, 024302 10.1063/1.2141951. 16422578

[ref13] León I.; Montero R.; Castaño F.; Longarte A.; Fernández J. A. Mass-Resolved Infrared Spectroscopy of Complexes without Chromophore by Nonresonant Femtosecond Ionization Detection. J. Phys. Chem. A 2012, 116, 6798–6803. 10.1021/jp303937h. 22639969

[ref14] León I.; Montero R.; Longarte A.; Fernández J. A. IR mass-resolved spectroscopy of complexes without chromophore: Cyclohexanol·(H_2_O)_*n*_, *n* = 1–3 and cyclohexanol dimer. J. Chem. Phys. 2013, 139, 174312 10.1063/1.4827110. 24206303

[ref15] Montero R.; León I.; Fernández J. A.; Longarte A. Femtosecond Excited State Dynamics of Size Selected Neutral Molecular Clusters. J. Phys. Chem. Lett. 2016, 7, 2797–2802. 10.1021/acs.jpclett.6b00997. 27388417

[ref16] Montero R.; Lamas I.; León I.; Fernández J. A.; Longarte A. Excited state dynamics of aniline homoclusters. Phys. Chem. Chem. Phys. 2019, 21, 3098–3105. 10.1039/C8CP06416D. 30672912

[ref17] Matsuda Y.; Mikami N.; Fujii A. Vibrational spectroscopy of size-selected neutral and cationic clusters combined with vacuum-ultraviolet one-photon ionization detection. Phys. Chem. Chem. Phys. 2009, 11, 1279–1290. 10.1039/b815257h. 19224026

[ref18] Nosenko Y.; Kunitski M.; Riehn C.; Harbach P. H. P.; Dreuw A.; Brutschy B. The structure of adenine monohydrates studied by femtosecond multiphoton ionization detected IR spectroscopy and quantum chemical calculations. Phys. Chem. Chem. Phys. 2010, 12, 863–870. 10.1039/B914236C. 20066371

[ref19] Mukhopadhyay A.; Cole W. T. S.; Saykally R. J. The water dimer I: Experimental characterization. Chem. Phys. Lett. 2015, 633, 13–26. 10.1016/j.cplett.2015.04.016.

[ref20] Van Thiel M.; Becker E. D.; Pimentel G. C. Infrared Studies of Hydrogen Bonding of Water by the Matrix Isolation Technique. J. Chem. Phys. 1957, 27, 486–490. 10.1063/1.1743753.

[ref21] Shinohara H.; Nishi N.; Washida N. Photoionization of water clusters at 11.83 eV: Observation of unprotonated cluster ions (H_2_O)^+^_n_ (2 ≤ *n* ≤ 10). J. Chem. Phys. 1986, 84, 5561–5567. 10.1063/1.449914.

[ref22] Shiromaru H.; Shinohara H.; Washida N.; Yoo H.; Kimura K. Synchrotron radiation measurements of appearance potentials for (H_2_O)_2_^+^, (H_2_O)_3_^+^,(H_2_O)_2_H^+^ and (H_2_O)_3_H^+^ in supersonic jets. Chem. Phys. Lett. 1987, 141, 7–11. 10.1016/0009-2614(87)80082-9.

[ref23] Radi P. P.; Beaud P.; Franzke D.; Frey H.-M.; Gerber T.; Mischler B.; Tzannis A.-P. Femtosecond photoionization of (H_2_O)_n_ and (D_2_O)_n_ clusters. J. Chem. Phys. 1999, 111, 512–518. 10.1063/1.479330.

[ref24] Dong F.; Heinbuch S.; Rocca J. J.; Bernstein E. R. Dynamics and fragmentation of van der Waals clusters: (H_2_O)_n_, (CH_3_OH)_n_, and (NH_3_)_n_ upon ionization by a 26.5eV soft x-ray laser. J. Chem. Phys. 2006, 124, 224319 10.1063/1.2202314. 16784286

[ref25] Belau L.; Wilson K. R.; Leone S. R.; Ahmed M. Vacuum Ultraviolet (VUV) Photoionization of Small Water Clusters. J. Phys. Chem. A 2007, 111, 10075–10083. 10.1021/jp075263v. 17715907

[ref26] Page R. H.; Frey J. G.; Shen Y. R.; Lee Y. T. Infrared Predissociation Spectra of Water Dimer in A Supersonic Molecular-Beam. Chem. Phys. Lett. 1984, 106, 373–376. 10.1016/0009-2614(84)85320-8.

[ref27] Coker D. F.; Miller R. E.; Watts R. O. The Infrared Predissociation Spectra of Water Clusters. J. Chem. Phys. 1985, 82, 3554–3562. 10.1063/1.448935.

[ref28] Huisken F.; Kaloudis M.; Kulcke A. Infrared spectroscopy of small size-selected water clusters. J. Chem. Phys. 1996, 104, 17–25. 10.1063/1.470871.

[ref29] Kuyanov-Prozument K.; Choi M. Y.; Vilesov A. F. Spectrum and infrared intensities of OH-stretching bands of water dimers. J. Chem. Phys. 2010, 132, 014304 10.1063/1.3276459. 20078158

[ref30] Otto K. E.; Xue Z.; Zielke P.; Suhm M. A. The Raman spectrum of isolated water clusters. Phys. Chem. Chem. Phys. 2014, 16, 9849–9858. 10.1039/c3cp54272f. 24398903

[ref31] Vernon M. F.; Krajnovich D. J.; Kwok H. S.; Lisy J. M.; Shen Y. R.; Lee Y. T. Infrared vibrational predissociation spectroscopy of water clusters by the crossed laser-molecular beam technique. J. Chem. Phys. 1982, 77, 47–57. 10.1063/1.443631.

[ref32] Huang Z. S.; Miller R. E. High-resolution near-infrared spectroscopy of water dimer. J. Chem. Phys. 1989, 91, 6613–6631. 10.1063/1.457380.

[ref33] Zhang B.; Yu Y.; Zhang Z.; Zhang Y.; Jiang S.; Li Q.; Yang S.; Hu H.; Zhang W.; Dai D.; et al. Infrared Spectroscopy of Neutral Water Dimer Based on a Tunable Vacuum Ultraviolet Free Electron Laser. J. Phys. Chem. Lett. 2020, 11, 851–855. 10.1021/acs.jpclett.9b03683. 31944117

[ref34] Pribble R. N.; Zwier T. S. Size-Specific Infrared-Spectra of Benzene-(H_2_O)_N_ Clusters (N = 1 Through 7) - Evidence for Noncyclic (H_2_O)_N_ Structures. Science 1994, 265, 75–79. 10.1126/science.265.5168.75. 17774690

[ref35] Tabor D. P.; Kusaka R.; Walsh P. S.; Zwier T. S.; Sibert E. L. 3rd Local Mode Approach to OH Stretch Spectra of Benzene-(H_2_O)_n_ Clusters, n = 2–7. J. Phys. Chem. A 2015, 119, 9917–9930. 10.1021/acs.jpca.5b06954. 26340135

[ref36] Burnham C. J.; Xantheas S. S.; Miller M. A.; Applegate B. E.; Miller R. E. The formation of cyclic water complexes by sequential ring insertion: Experiment and theory. J. Chem. Phys. 2002, 117, 1109–1122. 10.1063/1.1483259.

[ref37] Moudens A.; Georges R.; Goubet M.; Makarewicz J.; Lokshtanov S. E.; Vigasin A. A. Direct absorption spectroscopy of water clusters formed in a continuous slit nozzle expansion. J. Chem. Phys. 2009, 131, 204312 10.1063/1.3264576. 19947685

[ref38] Fajardo M. E.; Tam S. Observation of the cyclic water hexamer in solid parahydrogen. J. Chem. Phys. 2001, 115, 6807–6810. 10.1063/1.1410940.

[ref39] Ceponkus J.; Nelander B. Water Dimer in Solid Neon. Far-Infrared Spectrum. J. Phys. Chem. A 2004, 108, 6499–6502. 10.1021/jp049288v.

[ref40] Fröchtenicht R.; Kaloudis M.; Koch M.; Huisken F. Vibrational spectroscopy of small water complexes embedded in large liquid helium clusters. J. Chem. Phys. 1996, 105, 6128–6140. 10.1063/1.472472.

[ref41] Ng C. Y.; Trevor D. J.; Tiedemann P. W.; Ceyer S. T.; Kronebusch P. L.; Mahan B. H.; Lee Y. T. Photoionization of dimeric polyatomic molecules: Proton affinities of H_2_O and HF. J. Chem. Phys. 1977, 67, 4235–4237. 10.1063/1.435404.

[ref42] Kaiser E.; de Vries J.; Steger H.; Menzel C.; Kamke W.; Hertel I. V. Fragmentation dynamics of ammonia cluster ions after single photon ionization. Z. Phys. D: At., Mol. Clusters 1991, 20, 193–196. 10.1007/BF01543971.

[ref43] Martrenchard S.; Grégoire G.; Dedonder-Lardeux C.; Jouvet C.; Solgadi D. Proton transfer mechanism in the ionic methanol dimer. PhysChemComm 1999, 2, 15–19. 10.1039/A903329G.

[ref44] Zhang B.; Yu Y.; Zhang Y.; Jiang S.; Li Q.; Hu H.; Li G.; Zhao Z.; Wang C.; Xie H.; et al. Infrared spectroscopy of neutral water clusters at finite temperature: Evidence for a noncyclic pentamer. Proc. Natl. Acad. Sci. U. S. A. 2020, 117, 15423 10.1073/pnas.2000601117. 32541029 PMC7355007

[ref45] Mizuse K.; Fujii A. Characterization of a Solvent-Separated Ion-Radical Pair in Cationized Water Networks: Infrared Photodissociation and Ar-Attachment Experiments for Water Cluster Radical Cations (H_2_O)_n_^+^ (n = 3–8). J. Phys. Chem. A 2013, 117, 929–938. 10.1021/jp311909h. 23330841

[ref46] Tang M.; Hu C.; Lv Z.; Chen X.; Cai L. Ab Initio Study of Ionized Water Radical Cation (H_2_O)_8_^+^ in Combination with the Particle Swarm Optimization Method. J. Phys. Chem. A 2016, 120, 9489–9499. 10.1021/acs.jpca.6b09866. 27934325

[ref47] Jongma R. T.; Huang Y.; Shi S.; Wodtke A. M. Rapid Evaporative Cooling Suppresses Fragmentation in Mass Spectrometry: Synthesis of “Unprotonated” Water Cluster Ions. J. Phys. Chem. A 1998, 102, 8847–8854. 10.1021/jp983366v.

[ref48] Richard-Viard M.; Delboulbé A.; Vervloet M. Experimental study of the dissociation of selected internal energy ions produced in low quantities: application to N_2_O^+^ ions in the Franck-Condon gap and to small ionic water clusters. Chem. Phys. 1996, 209, 159–167. 10.1016/0301-0104(96)00164-4.

